# Medication Errors in Saudi Arabian Hospital Settings: A Systematic Review

**DOI:** 10.3390/medicina60091411

**Published:** 2024-08-29

**Authors:** Mansour Tobaiqy, Katie MacLure

**Affiliations:** 1 Department of Basic Medical Sciences, College of Medicine, University of Jeddah, Jeddah P.O. Box 45311, Saudi Arabia; 2 NHS Scotland, Aberdeen AB10 1AB, UK; katie.maclure@nhs.scot

**Keywords:** medication errors, medication safety, improvement strategy, prevention strategy, hospital, Saudi Arabia

## Abstract

*Background and Objectives:* Medication errors significantly impact patient safety, potentially causing adverse drug events (ADEs), increasing morbidity and mortality and prolonging hospital stays. This systematic review aimed to identify common medication errors in Saudi hospitals, their contributing factors, and effective prevention strategies. *Materials and Methods:* Following PRISMA-P guidelines, a comprehensive review of the literature published after 2019 was conducted. Inclusion criteria focused on peer-reviewed articles in English addressing medication errors in Saudi hospitals. Exclusion criteria eliminated reviews, opinion pieces, and non-peer-reviewed sources. A narrative synthesis identified common themes, and a descriptive analysis organized the data. *Results:* Searches yielded 22 articles from Embase (n = 4), PubMed (n = 10), and Web of Science (n = 8). After removing duplicates and one review article, twelve studies remained. Hand-searching references added 16 more, totaling 28 articles. Of the 28 included studies, 20 (71.4%) reported the types of medication errors observed. Wrong dose and improper dose errors are among the most frequently reported across multiple studies, while prescribing errors remain consistently high, indicating a critical area for intervention. Although less frequent, omission errors still hold significance. *Conclusions:* This review emphasizes the importance of comprehensive, proactive approaches to preventing medication errors. Integrating evidence-based strategies, fostering a safety culture, and continuously monitoring and evaluating interventions can significantly enhance medication safety and improve patient outcomes in Saudi Arabian hospitals.

## 1. Introduction

Medication errors are any preventable events that can cause inappropriate medication use or patient harm when the medicine is controlled by a healthcare professional, patient, or consumer. These errors can occur during prescribing, transcribing, dispensing, administering, and monitoring stages [1]. Medication errors lead to increased morbidity, mortality, and prolonged hospital stays. These errors can result in severe health complications, including allergic reactions, organ failure, and even death [2,3]. The implications of medication errors are profound, where in addition to increasing morbidity and mortality rates, these errors often lead to prolonged hospital stays [4].

The economic impact of medication errors is substantial both globally and in Saudi Arabia. This includes costs related to additional treatments, hospital re-admissions, legal fees, and lost productivity [5]. In Saudi Arabia, preventable medication errors significantly strain the healthcare system’s financial resources, although exact national figures are scarce [6,7]. 

The World Health Organization (WHO) initiated the third global patient safety challenge in 2017, targeting a 50% reduction in severe, avoidable medication-related harm over five years [8]. This initiative emphasized the worldwide impact of medication errors and recommended strategic interventions, including technologies, for countries to create and enforce national medication safety programs (WHO, 2017) [8,9,10].

Several studies have been conducted in Saudi Arabia to investigate the incidence of medication errors, their types, and frequency [3,11,12,13,14]. Al-Dhawailie (2011) explored medication errors in a pediatric hospital setting in Saudi Arabia [11]. This study identified high rates of medication errors, particularly in dosing and administration, emphasizing the importance of targeted interventions in pediatric care settings and reflecting studies conducted outside of Saudi Arabia [15,16]. 

Furthermore, several studies have reported the prevalence and nature of medication errors in Saudi hospitals, in addition to the high incidence of medication errors, particularly in the prescribing and administration stages, and the potential for clinical pharmacists to significantly reduce medication error rates [1,2,3,4,5,6,7,8,9,10,11,12,13,14,15,16,17]. The key barriers to reporting included a lack of awareness of the reporting policy, workload and time constraints, and the unavailability of reporting forms [12,13]. More recently, Abu Esba et al. (2018) investigated the impact of medication reconciliation at hospital admission on reducing medication errors. The study found that medication reconciliation significantly reduced errors at transitions of care, recommending the implementation of such processes in Saudi hospitals to enhance patient safety [3]. 

A year later, a 2019 systematic review across Middle Eastern countries considered the prevalence, nature, severity, and contributory factors of medication errors in hospitalized patients [17]. Medication errors were classified against Reason’s Causation Model [18]. The 10 Saudi Arabian articles out of the 50 studies identified the following:

- Active failures (slips—look-alike sound-alike medications, memory lapses; lapse—dispensing wrong drug, faulty dose checking; mistake—wrong packaging, preparation error; violation—poor adherence to protocol, breaking hospital rules);
- Error-producing conditions (miscommunication of drug orders; illegible prescription or records; wrong medication preparation by pharmacists; lack of knowledge; poor communication; lack of patient information);
- Latent failures (lack of educational activities; performance deficit; pharmacists not available 24 h a day; low staffing; poor drug stocking and delivery).

Further, Thomas et al. (2019) recommended that “Policy makers, leaders, practitioners and other relevant stakeholders must continue working towards minimizing the key-identified contributory factors where possible” [17].

Findings from these collective articles indicate a need to review and improve medication error reporting systems in Saudi Arabia to enhance health professional awareness and foster a better, more inclusive, less blame-focused culture supported by technology for enhanced reporting [1,2,3,4,5,6,7,8,9,10,11,12,13,14,15,16,17,18]. Of note, medication errors remain critical in Saudi Arabian healthcare facilities, even with robust health systems and preventive measures. Despite efforts to mitigate these incidents, they still threaten patient safety [11,12,13,14]. It is crucial to address this issue and assess the effectiveness of current prevention and management strategies [11,12,13,14]. This research aimed to identify the most common types of medication errors reported in Saudi hospitals, the factors contributing to those errors, and the strategies that have proven effective in preventing or improving them.

## 2. Materials and Methods

Following the PRISMA-P guidance for protocol development [19,20], a comprehensive review and analysis of the published literature on medication errors in hospital settings in Saudi Arabia was conducted. A protocol was registered with PROSPERO [21].

### 2.1. Inclusion Criteria

The inclusion criteria focused on peer-reviewed articles written in English and published after 2019, when Thomas et al. (2019) published their systematic review on the same topic but covering the wider Middle East [17]. The articles retrieved needed to specifically address medication errors in hospital settings within Saudi Arabia. Exclusion criteria were review articles, opinion pieces, and non-peer-reviewed sources.

### 2.2. Search Strategy

The search strategy utilized PubMed, Web of Science, and Embase databases to gather relevant articles. The same Boolean search string including wildcards (*) was applied to each database as follows:

(“medication errors” OR “medication safety”) AND (“prescribing” OR “dispensing” OR “administr*” OR “monitor*”) AND ((interven* OR improv* OR prevent*) AND strateg*) AND “Saudi Arabia” AND “hospital”.

Each article was screened independently for inclusion by both authors and then consensus was reached. Reference lists were hand-searched for any additional articles, published since 2019, matching the search criteria.

### 2.3. Data Extraction

For data extraction, a detailed form was designed to capture essential information from each study. This included study characteristics such as the study type, population, hospital type, location of errors, medicines involved, outcome and severity, incidence/prevalence, contributing factors, preventive or improvement strategies, outcomes, and effectiveness of strategies. 

### 2.4. Quality Assessment

Each of the included studies was subject to critical appraisal using the MMAT tool (Hong et al., 2018) appropriate to the study design [22].

### 2.5. Data Analysis

Given the likelihood of a wide range of study types, the planned data analysis involved a narrative synthesis to identify common themes and trends in the types of errors, their causes, and prevention and improvement strategies. Descriptive analysis was conducted to organize and code the data, utilizing Minitab 17 for statistical analysis. This approach allowed for a comprehensive understanding of medication errors in Saudi Arabian hospitals and the effectiveness of prevention and improvement strategies.

## 3. Results

**Figure 1:**
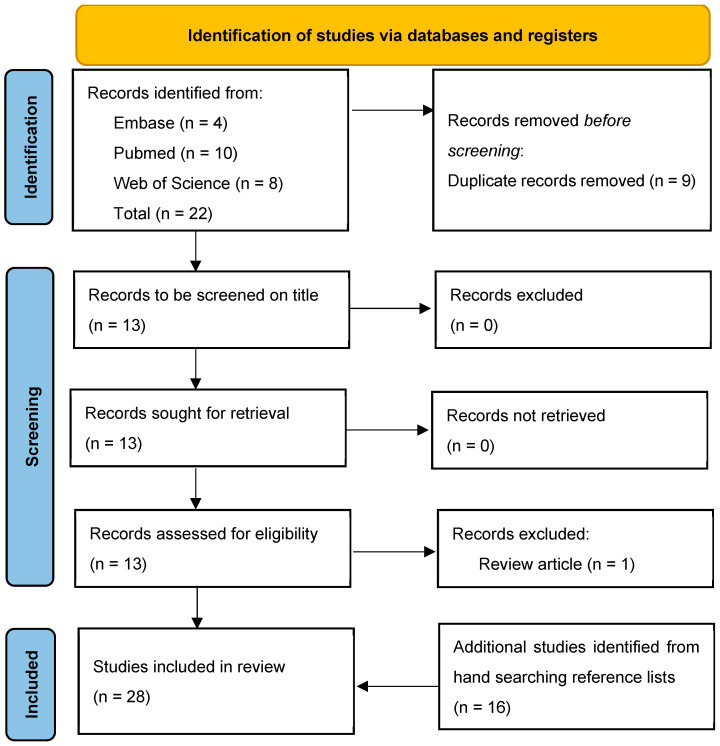
PRISMA flow diagram.

As shown in the PRISMA Flow Diagram [19], Figure 1, searches returned articles from Embase (n = 4), PubMed (n = 10), and Web of Science (n = 8), giving a total of 22 articles which, after duplicates (n = 9) were removed, reduced to 13 studies. One study was removed as it was a review article. Hand-searching the reference lists of each of the 12 provided an additional 13 articles for inclusion, yielding 25 articles for inclusion in this review.

The 28 studies included in this review comprised retrospective observational studies (n = 17, 60.7%), cross-sectional studies (n = 6, 21.4%), qualitative studies (n = 2, 7.1%), prospective observational studies (n = 2, 7.1%), and quality improvement projects (n = 1, 3.6%) [23,24,25,26,27,28,29,30,31,32,33,34,35,36,37,38,39,40,41,42,43,44,45,46,47,48,49,50].

### 3.1. Types of Medication Errors

Of the 28 included studies, 20 (71.4%) reported the types of medication errors observed, as per Table 1 [23,27,29,32,34,35,36,37,38,39,40,41,42,43,44,46,47,48,49,50]. Wrong dose and improper dose errors were among the most frequently reported across multiple studies, while prescribing errors remained consistently high, indicating a critical area for intervention [23,27,29,32,37,38,39,41,42,46]. Although less frequent, omission errors still held significance [23,43,44]. Errors related to incomplete orders were particularly prominent in studies on order entries [29,43]. Dispensing and administration errors are still noteworthy, though they are less commonly reported than prescribing errors [24,31,32,36,44,46]. Specific issues such as therapeutic duplication and incorrect dilution highlight potential areas for focused improvements [27,34,35,40,42,44,46]. Additionally, frequency and dosing schedule errors emphasize the importance of accurate scheduling in medication administration [27,29,38,42]. Furthermore, three studies reported on the prescribing and use of potentially inappropriate medications (PIMs) [48,49,50].

### 3.2. Strategies to Reduce Medication Errors

Numerous studies have proposed strategies to reduce medication errors and improve patient safety in Saudi Arabia [23,24,25,26,27,28,29,30,31,32,33,34,35,36,37,38,39,40,41,42,43,44,45,46,47,48,49,50].

The studies in this systematic review suggest various multifaceted approaches to minimize medication errors, as detailed in Table 1 [23,24,25,26,27,28,29,30,31,32,33,34,35,36,37,38,39,40,41,42,43,44,45,46,47,48,49,50]. These include awareness campaigns, closed-loop medication administration systems, independent double-check simulations, pediatric drug library utilization, and electronic order sets [23,24,25,26,27,28,29,30,31,32,33,34,35,36,37,38,39,40,41,42,43,44,45,46,47,48,49,50]. Cross-sectional studies emphasize the importance of effective communication among healthcare professionals and the benefits of automated drug dispensing systems [24,25,31,33,34,45]. Continuous education and training for healthcare professionals, especially nurses, are also crucial for reducing errors [34,45]. Additionally, three studies on potentially inappropriate medication prescribing and use recommend strategies such as regular medication therapy management, continuous reviews, prescription monitoring, and an anticoagulation stewardship initiative to enhance patient safety [51,52,53]. Prospective studies emphasize the importance of standardizing the ratio of critical care pharmacists (CCPs) to patients and implementing technology to optimize pharmacy services [25,28]. Retrospective observational studies reveal a high prevalence of medication errors and underscore the need for comprehensive documentation and continuous monitoring [26,27,29,32,35,37,38,39,41,42,43,44,46,47,48,49,50]. Electronic prescribing systems and the involvement of clinical pharmacists are crucial in reducing errors [15,26,37,38,39]. Routine pharmacist reviews and targeted interventions during high-risk times are essential [31,48]. Practical training for new doctors, educational interventions, and incorporating pediatric-specific information into computerized provider order entry (CPOE) systems further help to reduce medication errors [23,24,25,26,27,28,29,30,31,32,33,34,35,36,37,38,39,40,41,42,43,44,45,46,47,48,49,50]. 

## 4. Discussion

This systematic review analyzed 28 studies from Saudi Arabia (2019–2024) on medication errors [23,24,25,26,27,28,29,30,31,32,33,34,35,36,37,38,39,40,41,42,43,44,45,46,47,48,49,50]. Two-thirds of included studies reported types of errors, with wrong and improper doses being the most common [23,27,29,32,37,38,39,41,42,46]. Prescribing errors remained consistently high, indicating a need for intervention, whereas omission errors, though less frequent, are still significant [23,24,31,32,36,43,44,46]. The review highlights strategies to improve patient safety, such as quality improvement projects, technological interventions (automated drug dispensing and electronic prescribing systems), and standardized procedures [23,24,25,26,31,33,34,37,39,45]. Continuous professional training for healthcare professionals is essential [34,35].

Highlighting the need for targeted interventions during night shifts and weekdays, when errors tend to increase, this review underscores the pivotal role of clinical pharmacists in managing medications and preventing errors [41,42]. It also emphasized the importance of effective communication and coordinated care among healthcare professionals in reducing errors [24,30].

The systematic review underscores the effectiveness of multifaceted strategies, technology adoption, and the importance of continuous education. It identifies research gaps and offers evidence-based recommendations for improving medication safety, providing valuable insights for healthcare institutions and policymakers [23,24,25,26,27,28,29,30,31,32,33,34,35,36,37,38,39,40,41,42,43,44,45,46,47,48,49,50].

Three studies in this review reported on potentially inappropriate medication (PIM) prescribing and use [48,49,50]. PIM prescribing and use is a significant medication safety issue associated with adverse outcomes such as worsened health self-assessment, increased frailty, higher incidence of recurrent falls, depression, higher hospital admission rates, more ambulatory medical consultations, and increased medication prescriptions [48,49,50].

A systematic review and meta-analysis of 94 articles involving 371.2 million older participants found a 36.7% prevalence of PIM use, which has increased over the past two decades [52]. Jabri et al. (2023) found that 57% of older adults had at least one PIM in a year, with common PIMs including atorvastatin, metformin, aspirin, pantoprazole, and cholecalciferol [53]. These findings highlight the need for global healthcare reforms, improved drug safety, optimized prescriptions, and de-prescribing strategies for older patients [53]. This systematic review highlights the effectiveness of multifaceted quality initiatives in reducing medication errors in the PICU, as demonstrated by Ghezaywi et al. (2024) [23]. Similar findings by Berwick (2006) emphasize the importance of systemic changes and safety protocols [54]. Integrating closed-loop medication administration, independent double-checks, and electronic order sets aligns with successful quality improvement projects [23,55]. Cross-sectional studies reveal critical insights into medication errors across healthcare settings [24,25,31,33,34,45]. Alhur et al. (2024) stress the importance of communication among healthcare professionals, consistent with Sutcliffe’s findings on communication failures leading to errors [56]. Alanazi et al. (2022) highlight the benefits of automated drug-dispensing systems, supported by Poon (2010), who showed error reductions with barcode electronic systems [31,57].

Continuous education and training are crucial, as emphasized by Alotaibi et al. (2022), Alsulami et al. (2019), Manias et al. (2005), and Kohn et al. (2000) [34,45,58,59]. Ismail et al. (2023) underscore the need for standardized ratios of critical care pharmacists (CCPs) to patients and optimized pharmacy services through technology [25]. Bond et al. (2001) supports this by showing that increased pharmacist staffing reduces error rates [60]. Technological interventions like electronic health records and computerized physician order entry systems enhance medication safety [61].

Retrospective observational studies by Assiri et al. (2023), Alzaagi et al. (2023), and Alhossan et al. (2023) reveal high medication error prevalence, echoing Gandhi et al. (2003) findings of common errors in outpatient and inpatient settings [26,27,29,62]. Continuous monitoring and comprehensive documentation are crucial for error prevention, as Leape et al. (1998) emphasized [63]. Egunsola et al. (2021) and Alzahrani et al. (2021) discuss the role of electronic prescribing systems and clinical pharmacists, supported by Kaushal et al. (2001) and Bates et al. (1995) [15,38,39,64]. Alwadie et al. (2021) highlights routine pharmacist reviews, consistent with Bond et al. (2002) findings on the effectiveness of pharmacist-led interventions [60,65]. Aljuaid et al. (2021) and Alharaibi et al. (2021) note higher error rates during night shifts and weekdays, suggesting targeted interventions, in line with Lockley et al. (2004), who found that resident work-hour limitations reduce fatigue-related errors [41,42,66].

Of note, no single method can eliminate medication errors, but practicing vigilance and open communication among healthcare providers can reduce medication errors [67,68]. A study revealed that Foundation Year 1 (FY1) doctors believe their prescribing and safe medication use training needs improvement, raising patient safety concerns [69]. While education and training are crucial, patient safety also depends on support from a multidisciplinary team, including clinical pharmacists, trained nurses, and new technology, such as electronic prescribing systems with decision support [70]. Additionally, a collaborative approach, emphasizing accurate medication reconciliation, precise prescriptions, and standardized protocols, enhances safety by sharing information and resolving conflicts [67,68]. Training strategies such as simulation-based learning and interprofessional workshops improve decision making, teamwork, and communication, reducing errors [67,68,69,70].

### 4.1. Strength and Limitations

This systematic review boasts several strengths, including a comprehensive analysis that encompasses diverse study types, large sample sizes, and various healthcare settings, enhancing the generalizability of its findings. It effectively identifies common contributing factors to medication errors and provides evidence-based recommendations, making it valuable for developing targeted interventions. However, the review also has limitations, such as the heterogeneity of included studies, potential publication bias, varying study quality, and a reliance on cross-sectional or retrospective data, which limit the ability to assess longer term impacts (Table 1). Additionally, its focus on Saudi Arabian healthcare settings may not be directly applicable to other countries. Despite these limitations, the review’s broad scope and detailed insights contribute significantly to understanding and addressing medication errors.

### 4.2. Future Research

To build on the findings of this review, future research should focus on longitudinal and multi-center studies, standardization of study designs, evaluation of technological interventions, and patient involvement. Addressing these areas can enhance the understanding of medication errors and contribute to the development of more effective strategies for improving patient safety in healthcare settings. Comparative studies across healthcare settings and involving patients in the medication process will help identify universally effective and safe medical practices. Investigating the inappropriate use of medicine in the pediatric and geriatric population is essential to addressing their vulnerabilities and improving patient safety in this demographic.

## 5. Conclusions

This analysis of 28 studies on medication errors conducted in Saudi Arabia highlights wrong doses, improper doses, and prescribing errors as the most frequent, underscoring a critical need for intervention. Strategies to reduce these errors include awareness campaigns, closed-loop medication systems, independent double-checks, and electronic order sets. Effective communication among healthcare professionals, automated dispensing systems, continuous education, and regular pharmacist reviews are essential. Addressing potentially inappropriate medications through regular reviews and stewardship initiatives is also crucial. Implementing technology like electronic prescribing and optimizing pharmacist–patient ratios in critical care are vital strategies. Practical training for new doctors and incorporating pediatric-specific information into systems further help reduce medication errors.

## Figures and Tables

**Table 1:** Data extraction table from included studies (n = 28) [23,24,25,26,27,28,29,30,31,32,33,34,35,36,37,38,39,40,41,42,43,44,45,46,47,48,49,50].

Author, Year, Study Type, Reference	Population and Hospital Type	Location of Medication Errors, Medicines Involved, Outcome, and Severity of Medication Errors	Incidence/Prevalence, Types of Medication Errors, and Contributing Factors	Preventive or Improvement Strategies Implemented to Reduce Medication Errors	Outcomes and Effectiveness of These Strategies	Limitations/Biases
Ghezaywi et al. (2024) Quality Improvement Project [23]	1000 patients Pediatric Intensive Care Unit (PICU).	Hospital’s electronic medical record and nursing information systems	49 medication errors: wrong time errors (n = 23), improper dose errors (n = 8), omission errors (n = 6), prescribing errors (n = 6), wrong medication preparation errors (n = 2), wrong administration technique errors (n = 2), and other medication errors (n = 2).	-Awareness campaign; -Reinforcement of closed-loop medication administration system utilization; -Independent double-check simulations; -Pediatric drug library utilization and electronic order sets.	The study reported zero medication errors per 1000 patient days in the first quarter of 2022.	Reliance on self-reported data and direct observation may introduces potential reporting and observer bias. A single PICU at one hospital limits the generalizability of findings to other healthcare settings. The potential variability in the effectiveness of interventions across different PICU contexts.
Alhur et al. (2024) Cross-Sectional Study [24]	1165 healthcare professionals. Female (n = 763, 65.49%). Various settings within Saudi Arabia hospitals, clinics, and pharmacies.	Healthcare workers	Prescription errors (n = 233, 52.70%), dispensing errors (n = 40, 16.74%), documentation errors (n = 34, 16.65%), monitoring errors (n = 24, 13.91%). Medications inappropriate for the elderly should be used with caution or avoided.	Improving the quality of communication among healthcare professionals.	Showing that transparent and constructive environments significantly reduce medication errors.	Self-reported data may lead to overestimation of communication efficacy and under-reporting of errors. The cross-sectional design limits causality and generalizability.
Ismail et al. (2023) Cross-Sectional Study [25]	23 CCPs. Hospitals in Saudi Arabia.	Critical care pharmacists	Having 1–2 CCPs per critically ill patient.	Need for standardizing CCP-to-patient ratios and implementing technology to optimize services.	Emphasizes the need for standardized CCP-to-patient ratios and additional support for CCPs to expand their services	Response bias may have occurred as respondents were more likely to participate if their pharmacy departments had established clinical pharmacy services.
Assiri et al. (2023) Retrospective Observational Study [26]	117 adult females (n = 64, 54.7%). Family medicine clinics in King Faisal Specialist Hospital and Research Center.	Pharmacists. Of 117 patients, (n = 9, 7.7%) experienced preventable adverse drug events. All pADE cases involved OTC medications and polypharmacy. Specific outcomes: asthma (2 cases), β-blocker (4 cases), and single cases for warfarin/INR, lithium/lithium level, new oral anticoagulant/antiplatelet, and aspirin/antiplatelet.	9 patients (7.7%) experienced pADE.	Need for a multi-center study on clinically important medication errors at prescribing and monitoring stages to develop quality improvement programs.	This study reports the period prevalence of patients with pADEs in family medicine clinics.	Limited generalizability due to the single-center study. Retrospective assessments were constrained by available data, and information biases may have influenced severity perception.
Alzaagi et al. (2023) Retrospective Observational Study [27]	3210 prescriptions Mina Al Wadi Hospital	Intensive care unit, emergency room, medical ward, and outpatient department. Of the 43 medication errors reported, 97.67% were near misses, while only 2.32% reached patients without causing injury.	There were 43 medication errors reported, with the following causes: lack of drug information (58.14%), wrong drugs (23.25%), workload issues (23.25%), frequency (18.60%), and medication issues (18.60%).	The study found that medication errors were in line with global standards, often preventable, and rarely reached pilgrims.	The most common error was incorrect medication, mainly due to lack of drug information.	Voluntary reporting at a single hospital likely leads to the under-reporting of medication errors. The study was conducted in a temporary healthcare setting during Hajj; thus, it lacks generalizability.
Alothmany et al. (2023) Qualitative Study [28]	8 interviews, male (n = 3), female (n = 5). Pharmacy department of a tertiary care hospital.	Pharmaceutical Services.		-Improving high-alert medication safety (85.7%); -Having antidotes available (75%); -Verifying sterile preparations (75%); -Maximizing barcode verification (12.5%).	Barriers included lack of knowledge, motivation, and environmental resources. Healthcare providers need knowledge, motivation, and resources to implement best practices effectively.	The study’s single-center design and small sample size (eight managers, with only five interviewed) may limit the generalizability of the findings, though all participants completed the survey.
Alhossan et al. (2023) Retrospective Observational Study [29]	A total of 9215 reports on ordering and prescribing errors were collected from 6 tertiary care hospitals in Riyadh, Saudi Arabia.	Errors occurred primarily in the ICU (n = 3919, 42.5%), followed by the NICU (n = 3207, 34.8%), CCU (n = 1336, 14.5%), and PICU (8.5%).	Out of 9215 medication orders, incomplete orders (n = 1928, 21%), lacking drug information (n = 1503, 16%), dosing schedule errors (n = 1290, 14%), duplicate drug class errors (n = 914, 10%), and wrong dose errors (n = 647, 7%).	-Improved medication errors documentation and continuous monitoring.	Emphasize the crucial role of inpatient pharmacists in detecting, reporting, and reducing prescription-related errors.	Including only prescription errors detected by inpatient pharmacists would likely underestimate the true incidence of medication errors. Emphasizing medication safety in the ICU and encouraging error reporting could further reduce these errors.
Al Rowily et al. (2023) Qualitative Study [30]	9 patients, female (n = 3), male (n = 6). Tertiary care hospitals.	Direct oral anticoagulants (DOACs).	Lack of knowledge on DOACs, difficulty accessing DOACs.	Develop and assess theory-based interventions to enhance patient knowledge and understanding shared decision making.	Effective communication, timely clinic access, availability of medications key to optimizing DOAC utilization safety.	The study’s results, drawn from a limited sample size, may not fully represent the broader patient population.
Alanazi et al. (2022) Cross-Sectional Study [31]	49 clerkship students, female (n = 25) and male (n = 24). Tertiary care hospitals.	Pharmacists.	Daily prescription errors exceeded 10 with automated drug dispensing system (ADDs) at 4.16% and traditional drug dispensing system at 2.11%.	To allocate more time for patient care.	ADDs effectively improved dispensing practices and medication reviews.	Potential selection bias. The study relied on self-reported prescription errors and high-alert medication dispensing rather than incident data analysis. Additionally, the small sample size may not reflect the broader pharmacist community’s views.
Almuqbil et al. (2022) Retrospective Observational Study [32]	A total of 243 drug information inquiries were evaluated. Tertiary hospital.	Enquires from pharmacists, physicians, and nurses. Antibiotics (13.6%) were the most common, followed by antihypertensive agents (11.1%), anticoagulants (8.5%), and anticonvulsants (6.8%).	N = 243. Prescribing errors (88.1%) were the most prevented by drug info specialists, followed by dispensing errors (4.5%). About half were near-misses (45.3%), with potential near-misses at 34.6%, only 20.2% were actual errors.	Pharmacists supplying evidence-based information to the inquirer.	This study highlights the role of drug information specialists in preventing medication errors and enhancing patient safety through evidence-based information.	The study’s small sample size from a single institution in Saudi Arabia’s capital may limit generalizability. Reliance on providers’ willingness to report errors might also lead to underestimations.
Alomi et al. (2022) Cross-Sectional Study [33]	Out of the 253 responding physicians’ female (n = 72, 60.50%). An electronic self-reported survey of physicians in Saudi Arabia.	Anticoagulants (33.33%) and NSAIDs (32.32%) most reported in medication errors. Sedatives (38.78%) and muscle relaxants (36.73%) were common in ADR cases. Antineoplastics (40.00%) and anti-seizure drugs (39.39%) frequently appeared in look-alike sound-alike issues.	Physicians’ medication safety cultures were insufficient and inconsistent.	Develop medication safety cultures.	It is recommended to revise medication safety culture guidelines and enhance education and training to improve knowledge and practices related to medication safety.	The study’s cross-sectional design involved mostly young doctors with limited experience.
Alotaibi et al. (2022) Cross-Sectional Study [34]	A total of 260 nurses participated, with males (n = 177, 68.1%), females (n = 83, 31.9%). KSMC in Riyadh, Saudi Arabia	Nurses.	35% of nurses reported no medication errors. 12.3% reported committing five errors, 11.5% who reported one error during their nursing career. Physicians’ illegible handwriting, incorrect drug dose prescriptions, nurse fatigue.	Medication errors should be documented through incident reports in a confidential, informative, and penalty-free system.	Continuous education and on-the-job training for nurses in medication administration are essential in hospitals.	Participants’ differing insights may be overlooked, and convenience sampling could lead to under-representation, limiting the generalizability of the findings.
Alyami et al. (2022) Retrospective Observational Study [35]	All medical records at King Khalid Hospital of Najran region, Saudi Arabia.	Healthcare professionals and departments. Antibiotics, anticoagulants, and antihypertensives.	There were 4860 medication errors: 21.2% involved overdose or underdose, 6.2% inappropriate dosage units, 6.1% therapeutic duplication. Ordering/prescribing/transcribing 66.9%, admin for 28.8%, preparing/dispense 2.9%.	Improving medication ordering, prescribing, and transcribing in hospital settings.	This study highlights common medication errors and provides useful tips for reducing these errors and enhancing patient safety.	The study’s results were drawn from a single hospital, limiting the findings’ generalizability. The study relies on data extracted from electronic medical records, which may miss undocumented or under-reported medication errors.
Eid et al. (2022) Prospective Observational Study [36]	A standard paper-based tool and digital stopwatch counted interruptions. Nurses’ sources, secondary tasks, and interruption impacts were considered. Tertiary teaching hospital.	Nurses.	A total of 87 medication-related events occurred, with 182 interruptions accounting for 90%. Interruptions were frequent during medication administration, often in corridors and patients’ rooms. Nurses, medical officers, and impediments were common sources. System failures were linked to clinical and procedural errors.	Using an Omnicell automatic dispensing machine and cabinets in each patient’s room. Wearing a “no-interruption zone” sign, can be tailored to the practice context to reduce interruptions.	90% of medication work events were interrupted, mostly during administration tasks.	The study’s limitations include convenience sampling, a small sample size, the Hawthorne effect, and data collection during the busy morning shift, limiting generalizability to other contexts.
Memon SI. (2022) Retrospective Observational Study [37]	253 medical records, male (n = 123) female (n = 130). A tertiary care teaching hospital in Riyadh, Saudi Arabia.	Admissions and inpatient treatment records.	Out of 248 incidents (98.2%), wrong doses (n = 118, 46.6%), duplicate therapies (n = 69, 27.33%), wrong medications (n = 16, 6.3%). Also, workplace violence and nursing practices.	Near misses should recognized as key targets for continuous quality improvement tools to reduce preoperative incidents in hospitals.	Organizational lapses were identified as critical targets for mitigating perioperative incidents in the hospital.	Near-miss data vary by hospital. A key limitation is the lack of classification based on immediate corrective actions taken.
Egunsola et al. (2021) Retrospective Observational Study [38]	109,382 drugs were ordered. Tertiary hospital in Saudi Arabia.	Healthcare professionals. Intravenous medicines (n = 2985, 32.7%), oral (n = 2199, 24.1%), inhalation (n = 2081, 22.8%). 0.4% reached the patient but caused no harm.	Out of 9123 medication errors: wrong frequency (39.1%), wrong drug (12.5%), wrong concentration or strength (12.4%), and wrong dose (11.1%).	Electronic prescribing system and integrated clinical pharmacists into patient care.	Medication errors are common in pediatric care, particularly with drugs like paracetamol and amoxicillin.	Under-reporting occurred due to fear, time constraints, lack of awareness, and disinterest. Root cause analyses were not conducted, limiting insights into MEs and hindering the development of preventive strategies.
Alzahrani et al. (2021) Retrospective Observational Study [39]	A total of 2564 pharmacist interventions related to medication prescribing errors. Tertiary teaching hospital.	Pharmacists. Anti-infectives for systemic use (49.2%) and alimentary tract and metabolism medications (18.2%).	Out of 2564 medication errors, 54.3% were wrong doses and 21.9% were unauthorized prescriptions.	Care coordination and patient safety prioritization through quality improvement initiatives are crucial at all levels of the healthcare system.	Medication errors were common, and pharmacist interventions were crucial in preventing potential patient harm.	The study, conducted in a single tertiary hospital, may have missed some PEs due to incomplete records. Additionally, variations in how pharmacists provided and reported interventions could affect the findings.
Alwadie et al. (2021) Prospective Observational Study [40]	14,144 pharmacists’ interventions were recorded. King Abdulaziz Medical City (KAMC) hospital in Jeddah.	Pharmacist. Perfusion solutions (41%), and antibacterial (35%).	Order entry errors occurred at a rate of 9.1%. The most frequent issue was incorrect dilution, accounting for 40.2% of cases (n = 972). This was followed by dose substitution at 27.7% (n = 665), and duplicate therapy at 10.3% (n = 248).	Routine pharmacist review of inpatient drug therapy is essential for ensuring the quality use of medicines.	Therapeutic Intervention Documentation (TID) holds significant potential to decrease drug-related problems.	Focusing on the most common interventions (93.4%) might overlook less frequent but highly significant interventions.
Aljuaid et al. (2021) Retrospective Observational Study [41]	Examining medication errors reported by healthcare practitioners January 2018 and December 2019. Primary, secondary, and tertiary care.	Healthcare practitioners.	Out of 2626 medication errors, 55% were prescribing errors, 14.1% were due to lack of availability, and 10.1% involved delayed medications. Medication errors were more likely to occur during night shift compared to the day shift.	The timing of medication errors is crucial for enhancing medication use and patient safety.	There is a statistically significant relationship between medication errors and the day of the week, with higher incidence during weekdays compared to weekends.	Voluntary reporting, influenced by healthcare providers’ awareness and fear of punitive actions, leads to under-reporting. Cultural differences among nurses also affect the reporting of administration errors.
Alharaibi et al. (2021) Retrospective Observational Study [42]	315,166 prescriptions were screened. Tertiary care.	Medical residents comprised the largest group (52%, n = 2577), followed by specialists (33%, n = 1629). Drugs for the alimentary tract and metabolism accounted for 1156 prescriptions (23.4%). Two-thirds of prescribing errors were harmless to patients.	There were 4934 prescribing errors (1.56%), improper dose (n = 1516, 30.7%), and incorrect frequency (n = 987, 20%). Prescribing errors were linked to inadequate documentation of clinical information.	Future studies should aim to test innovative measures to control these factors and assess their impact on reducing prescribing errors.	Insufficient documentation in electronic health records and the prescription of anti-infectives were the predominant factors predicting PEs.	The study is based on data from a single healthcare setting, which may limit generalizability. The study did not account for the total number of drugs per prescription, which could increase the likelihood of prescribing errors due to polypharmacy.
Kayamkani et al. (2020) Retrospective Observational Study [43]	130 patients Tertiary care hospital.	Healthcare. 27% involved minor drug–drug interactions, 9% were moderate interactions, and 2% were classified as serious drug–drug interactions.	28% of prescriptions were incomplete, with issues such as missing dose information (7%), inadequate directions (2.7%), and omission of treatment duration (3.5%).	Computerizing the medication process and providing a drug formulary in hospitals can serve as quick references for prescribers. Effective training of newly appointed doctors.	Pharmacists’ roles are crucial in detecting and correcting medication errors, requiring formal acknowledgment and incorporation into a routine monitoring.	The limited number of patient prescriptions from a single tertiary hospital reduces the study’s representativeness and generalizability.
Aseeri et al. (2020) Retrospective Observational Study [44]	Analysis was conducted on all reported medication error incidents. Tertiary care center.	Healthcare professionals. Chemotherapeutic agents accounted for 23.6%, anticoagulants for 7.5%, and opiates/narcotics for 4.8% of cases. Near misses (69.3%).	There were 624 medication errors in dispensing/administration, comprising 15% of cases. Further, 13% were due to incorrect dose, 8.9% involved the wrong patient, and 7.5% resulted from dose omission.	Prescribing oral liquid medications exclusively using the metric weight system (e.g., mg).	The hospital medication safety reporting program is a valuable tool for identifying system-based issues in medication management.	The reliance on voluntary reporting may lead to under-reporting or selective reporting of medication errors, introducing bias in the data. The study was conducted in one tertiary care center, so the findings may need to be more generalizable to other institutions or regions.
Alsulami et al. (2019) Cross-Sectional Study [45]	365 participants, 89.3% were female. Healthcare institution.	Pediatric, internal medicine, surgery, family medicine and obstetrics and gynecology	57.1% acknowledged it was their responsibility to report such errors. Poor performance, insufficient information, slips, and lapses.	All healthcare practitioners should undergo mandatory drug safety training, and error-detecting alarms and software should be utilized.	Strengthening error reporting with robust regulations and adequate training for healthcare personnel.	Recall bias of participants, social desirability bias and communication barriers between investigators and participants may have yielded imprecise data. The study was conducted at one tertiary care center; therefore, the study results cannot be generalized to the whole healthcare population.
Alshahrani et al. (2019) Retrospective Observational Study [46]	386 adult patients records were reviewed (>15 years old). Tertiary hospital.	Healthcare professional.	A total of 113 medication errors were recorded, comprising 112 prescribing errors and 1 dispensing error. Medication duplication (31.2%) was the most frequent issue, followed by missing patient-identifying information (25%).	Implementing educational interventions, such as workshops and continuous medical education, is crucial for minimizing and preventing medication errors.	Numerous medication errors have been identified with most being related to inpatient prescriptions.	This retrospective study was conducted at a single healthcare facility in the southern province of Saudi Arabia, limiting the generalizability of the results.
AlAzmi et al. (2019) Retrospective Observational Study [47]	657 pediatric patients: 387 males (58.9%). Hospitalised children at KAMC-J.	Pediatric wards and/or the emergency department. 15 minor (4.6%), 312 moderate (95.1%), and 1 severe (0.3%).	328 confirmed drug-related problems (DRPs): dosing problems accounted for 64.9% (n = 213/328) and drug choice problems for 32.9% (n = 108/328).	Incorporating a more pediatric-specific information into CPOE is essential to further reduce these problems and enhance the care.	The computerized physician order entry (CPOE) system has significantly reduced DRP incidence in children.	This study reflects the experience of a single institution. It also did not assess the outcomes of preventable DRPs or off-label medication use in pediatrics, areas that should be explored in future research.
Alhawassi et al. (2019) Retrospective Observational Study [48]	Review of medical records of 4073 older adults (56.8% female, 43.2% male) in ambulatory care clinics at a tertiary hospital.	Ambulatory care clinics. 57.6% of older adults had potentially inappropriate medications (PIMs) to be avoided, and 37.5% had PIMs that should be used with caution.	The most frequently prescribed PIMs were gastrointestinal agents (35.6%) and endocrine agents (34.3%).	Regular medication therapy management and continuous reviews can help reduce PIM usage.	Elderly patients may benefit from a multidisciplinary care model with pharmacist follow-up to assess and minimize inappropriate medications.	The findings of this study cannot be generalized to all older adults in Saudi Arabia, as it only included older patients from the ambulatory care clinics of a single tertiary hospital.
Alanazi et al. (2024) Retrospective Observational Study [49]	Review of PIMs in two hospitals involving 237 patients (50.8% male, 49.2% female).	Medical/surgical wards. The overall prevalence of PIMs was 29.2%, with 11% specifically to be avoided in elderly patients.	The most common PIM medications were proton pump inhibitors (44.41%), particularly among patients discharged from the surgical unit.	Prescription monitoring is recommended to prevent medication errors, particularly for patients on multiple medications.	Community-based studies are needed to confirm this trend and identify the factors causing PIM prescriptions.	The limitations include restricted access to additional factors like socioeconomic background and prescriber details. Over-the-counter PIMs, which are common in Saudi Arabia, may have led to an underestimation of PIM frequency among the elderly.
Sultan et al. (2023) Retrospective Observational Study [50]	Review of direct oral anticoagulant (DOAC) utilizations in a tertiary hospital with 778 patients.	Among 232 patients (29.8%), 41.7% had inappropriate maintenance doses, 37.97% had inappropriate initial doses, and 36.42% lacked laboratory monitoring.	40.8% received rivaroxaban, 31.02% received apixaban.	They recommended establishing and monitoring an anticoagulation stewardship initiative to improve medicines utilization.	The outcomes of these suggestions have yet to be determined.	A significant percentage of patients on DOACs were excluded due to incomplete data.

## Data Availability

Data that support the findings of this study are available from the corresponding author, upon reasonable request.
